# Supplementary material for: Resolving the full spectrum of human genome variation using Linked-Reads
Source: Genome Res. 2019 Apr;29(4):635–45. doi: 10.1101/gr.234443.118 (PMC6442396; doi:10.1101/gr.234443.118)
Supplement: Supplemental Material [file supp_gr.234443.118_Supplemental_longranger-2.2.zip › longranger-2.2.tar.bz2!/lr2.2-public/lib/stan/cmdstan/stan/doc/api/html/internal_8hpp_source.html]

Stan: src/stan/agrad/rev/internal.hpp Source File


|  |  |
| --- | --- |
|  | Stan  2.5.0  probability, sampling & optimization |


- Main Page
- Related Pages
- Namespaces
- Classes
- Files

- File List
- File Members

All Classes Namespaces Files Functions Variables Typedefs Enumerations Enumerator Friends Macros Pages

- src
- stan
- agrad
- rev

internal.hpp

Go to the documentation of this file.

1 #ifndef STAN\_\_AGRAD\_\_REV\_\_INTERNAL\_HPP

2 #define STAN\_\_AGRAD\_\_REV\_\_INTERNAL\_HPP

3

4 #include <stan/agrad/rev/internal/ddv\_vari.hpp>

5 #include <stan/agrad/rev/internal/dv\_vari.hpp>

6 #include <stan/agrad/rev/internal/dvd\_vari.hpp>

7 #include <stan/agrad/rev/internal/dvv\_vari.hpp>

8 #include <stan/agrad/rev/internal/matrix\_vari.hpp>

9 #include <stan/agrad/rev/internal/precomp\_v\_vari.hpp>

10 #include <stan/agrad/rev/internal/v\_vari.hpp>

11 #include <stan/agrad/rev/internal/vd\_vari.hpp>

12 #include <stan/agrad/rev/internal/vdd\_vari.hpp>

13 #include <stan/agrad/rev/internal/vdv\_vari.hpp>

14 #include <stan/agrad/rev/internal/vector\_vari.hpp>

15 #include <stan/agrad/rev/internal/vv\_vari.hpp>

16 #include <stan/agrad/rev/internal/vvd\_vari.hpp>

17 #include <stan/agrad/rev/internal/vvv\_vari.hpp>

18

19 #endif

vvd\_vari.hpp

precomp\_v\_vari.hpp

dv\_vari.hpp

dvd\_vari.hpp

matrix\_vari.hpp

vdd\_vari.hpp

vd\_vari.hpp

vector\_vari.hpp

v\_vari.hpp

vvv\_vari.hpp

dvv\_vari.hpp

vdv\_vari.hpp

vv\_vari.hpp

ddv\_vari.hpp


---

[ Stan Home Page ]

*© 2011–2014,
Stan Development Team.*
